# Complications des mutilations génitales féminines : épidémiologie et prise en charge des cas au Centre hospitalier universitaire (CHU) régional de Ouahigouya, Burkina Faso

**DOI:** 10.48327/mtsi.v4i1.2024.463

**Published:** 2024-01-04

**Authors:** Sansan Rodrigue SIB, Évelyne KOMBOÏGO, Moussa SANOGO, Issa OUÉDRAOGO, Alexandre TARNAGADA, Souleymane TRAORÉ, Charlemagne Marie Ragnag-Néwende OUÉDRAOGO

**Affiliations:** 1 Service de gynécologie obstétrique, CHU de Ouahigouya, Burkina Faso; 2 Département de Gynécologie obstétrique et de Médecine de la reproduction, CHU Sourô Sanou, Bobo Dioulasso, Burkina Faso; 3 Maternité Antonio Bruni, Centre médico-chirurgical pédiatrique Persis, Ouahigouya, Burkina Faso; 4 Département de gynécologie obstétrique, CHU de Bogodogo, Ouagadougou, Burkina Faso

**Keywords:** Mutilations génitales féminines, Complications, Séquelles d'excision, Rétrécissement vulvaire, Urocolpos, Hôpital, Ouahigouya, Burkina Faso, Afrique subsaharienne, Female genital mutilation, Complications, Sequelae of female genital mutilation, Vulvar stricture, Urocolpos, Hospital, Ouahigouya, Burkina Faso, Sub-Saharan Africa

## Abstract

**Introduction:**

Les mutilations génitales féminines sont encore fréquentes au Burkina Faso, malgré des décennies de lutte contre leur pratique. La région du Nord de ce pays connaît l'une des plus fortes prévalences de cette pratique sur le plan national. L'objectif de notre étude est de décrire les complications sanitaires des mutilations génitales féminines prises en charge dans l'hôpital de référence de cette région.

**Patientes et méthodes:**

Il s'est agi d'une étude rétrospective des données sur une période de 13 ans, du 15 septembre 2009 au 14 septembre 2022. Les patientes admises pour des complications génitales ou loco-régionales liées aux mutilations génitales ont été incluses. Les parturientes mutilées sans infibulation, victimes de déchirures vulvaires ou ayant bénéficié d'une épisiotomie n'ont pas été incluses.

**Résultats:**

Nous avons dénombré 204 patientes, soit 3,1 % des consultantes. La tranche d’âge de 15 à 20 ans a été la plus représentée (49,3 %), et les âges extrêmes étaient 15 mois et 31 ans. Les principaux motifs de consultation étaient le rétrécissement vulvaire, la dyspareunie, l'impossibilité des rapports sexuels, et la dysurie. Il s'agissait de complications en rapport avec une infibulation dans 81,8 % des cas et avec une mutilation de type II dans 18,2 %. La chirurgie a constitué 89,9 % des traitements et les traitements médicamenteux seuls 10,1 %. Aucune reconstruction du clitoris n'a été réalisée. L’évolution a été favorable dans tous les cas. Conclusion. Les complications locales et régionales des mutilations génitales sont multiples, mais heureusement leur prise en charge est de bon pronostic sur le plan anatomique. Les complications sur le plan psychologique restent cependant à être évaluées et prises en charge, dans notre contexte. La prise en charge de ces complications devrait être une occasion de sensibilisation de l'entourage pour l'abandon de la pratique.

## Abstract (en)

**Background:**

Female genital mutilation is still common in Burkina Faso, despite decades of struggle against its practice. The northern region of this country has one of the highest prevalence of this practice at the national level with 76% of women mutilated. The objective of our study was to describe the health complications of female genital mutilation treated in the referral hospital in this region.

**Patients and methods:**

This was a descriptive cross-sectional study with retrospective data collection over a 13-year period, from September 15, 2009 to September 14, 2022. Patients admitted for genital or loco-regional complications related to genital mutilation were included. Mutilated parturients without infibulation, victims of vulvar tears or who had undergone episiotomy were not included.

**Results:**

We recorded 204 patients, representing 3,1% of consultants, and an annual frequency of 15.7 cases. The ages of the victims ranged from 15 months to 31 years. The 15-20 age group was the most represented (49.3%). Victims were more likely to come from urban than rural areas. The main reasons for consultation were vulvar stricture, dyspareunia, impossibility of sexual intercourse, and dysuria. These were medium- and long-term complications of the mutilation. These complications were related to infibulation in 81.8% of cases and to type II mutilation in 18.2%. Surgery accounted for 89.9% of treatments, with drug treatments alone accounting for 10.1%. Deinfibulation was the most common surgical procedure. No clitoral reconstruction was performed. The outcome was favourable in all cases.

**Conclusion:**

There are many local and regional complications of genital mutilation, but fortunately their treatment has a good anatomical prognosis. However, psychological complications remain to be evaluated and managed in our context. The management of these complications should be an opportunity to raise awareness among the patients’ family circles to abandon the practice.

## Introduction

Selon l'Organisation mondiale de la santé (OMS), les mutilations génitales féminines recouvrent toutes les interventions incluant l'ablation partielle ou totale des organes génitaux externes de la femme ou toute autre lésion des organes génitaux féminins qui sont pratiquées pour des raisons non médicales. Ces mutilations ne présentent aucun avantage sur le plan de la santé. Elles constituent une atteinte aux droits des victimes [11], notamment les droits au meilleur état de santé possible, à l'intégrité physique, à l’égalité et la non-discrimination liée au genre, à une vie exempte de torture et de traitements cruels, inhumains ou dégradants et même dans certains cas, le droit à la vie.

Le Burkina Faso fait partie de la trentaine de pays dans le monde où elles sont fréquemment pratiquées. Dans ce pays, malgré de nombreuses actions de lutte, y compris le vote d'une loi en 1997 punissant leur pratique, elles sont encore courantes, avec cependant des variations de la prévalence en fonction de certains facteurs. Ainsi, la prévalence des femmes mutilées est moins importante dans les zones urbaines, dans les milieux socioéconomiques élevés, et dans certaines régions comme celle du Centre-Ouest. La région du Nord connaît l'une des prévalences les plus importantes de cette pratique sur le plan national [10]. Elle est également caractérisée par l'existence d'autres pratiques culturelles néfastes comme le lévirat, le mariage forcé, le mariage de jeunes adolescentes. De plus, les violences sexuelles y sont en recrudescence depuis 2015, du fait de l'insécurité liée aux groupes armés terroristes qui a désorganisé le tissu social et entraîné de nombreux déplacements de populations.

C'est dans ce contexte que de nombreuses jeunes filles et femmes ayant vécu la douleur, l'hémorragie et d'autres complications immédiates lors de la mutilation, continuent de souffrir d'autres séquelles souvent très graves liées à cette pratique. Nombre d'entre elles sont reçues au Centre hospitalier universitaire régional de Ouahigouya, le centre de référence de la région du Nord, en quête de soins. L'objectif de notre étude était de décrire les caractéristiques épidémiologiques et la prise en charge des complications liées aux mutilations génitales féminines dans l'hôpital de référence d'une région où cette pratique est encore très répandue.

## Patientes et méthodes

Il s'est agi d'une étude transversale descriptive avec une collecte de données rétrospective qui a été menée dans le service de gynécologie obstétrique du Centre hospitalier universitaire régional de Ouahigouya. Elle a concerné une période de 13 ans, du 15 septembre 2009 au 14 septembre 2022.

L’étude a concerné les complications génitales et loco-régionales liées aux mutilations génitales féminines.

Ainsi, ont été incluses dans l’étude les patientes qui, au décours d'une mutilation génitale, ont présenté à court, moyen ou long terme, une complication.

Les complications psychologiques telles que le syndrome de stress post-traumatique et les troubles liés au désir et au plaisir sexuels n'ont pas été incluses, de même que l’épisiotomie et les déchirures périnéales lors de l'accouchement chez les patientes porteuses d'une mutilation des types I et II (pas de rétrécissement vulvaire). En effet, les informations disponibles ne permettaient pas soit de les identifier, soit de les lier aux mutilations. Toutes les patientes répondant aux critères de sélection ont été retenues pour notre étude.

La classification des mutilations génitales féminines établie par l'OMS du 31 janvier 2023 [11] a été utilisée pour caractériser les types de mutilations rencontrés. Cette classification définit 4 types de mutilations :

- Type I : ablation partielle ou totale du gland clitoridien (petite partie externe et visible du clitoris et partie sensible des organes génitaux féminins) et/ou du prépuce/capuchon clitoridien (repli de peau qui entoure le clitoris).
- Type II : ablation partielle ou totale du gland clitoridien et des petites lèvres (replis internes de la vulve), avec ou sans excision des grandes lèvres (replis cutanés externes de la vulve).
- Type III : infibulation - rétrécissement de l'orifice vaginal par recouvrement, réalisé en sectionnant et en repositionnant les petites lèvres, ou les grandes lèvres, parfois par suture, avec ou sans ablation du prépuce/capuchon et gland clitoridiens (type I).
- Type IV : toutes les autres interventions néfastes au niveau des organes génitaux féminins à des fins non médicales, par exemple, piquer, percer, inciser, racler et cautériser les organes génitaux.

Ont été considérées comme traitement chirurgical, la désinfibulation, et l'excision de chéloïdes suivie de soins locaux et de traitements antalgiques.

Les traitements médicamenteux seuls désignaient les injections de corticoïdes sous les chéloïdes, les applications locales d'antiseptiques, la prise d'antibiotiques ou d'antalgiques par voie générale, sans traitement chirurgical préalable.

Les données ont été recueillies à partir des dossiers médicaux à l'aide d'une fiche de collecte de données.

L'analyse de ces données a été réalisée à l'aide du logiciel Epi Info 7.

## Résultats

### Fréquence

Pendant la période d’étude de 13 ans, le nombre moyen annuel de consultantes en gynécologie et en obstétrique était de 6 673. Nous avons colligé 204 patientes porteuses de complications liées à la pratique de mutilations génitales féminines, et répondant à nos critères de sélection, soit 15,7 cas par an et 3,1 % des consultantes.

### Âge des patientes

L’âge des patientes variait entre 15 mois et 31 ans. L’âge de 17 ans constituait la valeur modale. Les moins de 15 ans représentaient 31,4 % de la population. Le Tableau I présente la répartition des patientes selon les tranches d’âge.

**Tableau I:** Répartition par tranches d’âge des patientes prises en charge pour une complication de mutilation génitale féminine au CHU régional de Ouahigouya de 2009 à 2022 Age distribution of patients treated for complications of female genital mutilation at the Ouahigouya regional teaching hospital from 2009 to 2022

Âge (années)	Effectif	Pourcentage
[00 - 05[	10	4,9
[05 - 10 [	33	16,2
[10 - 15 [	21	10,3
[15 - 20 [	99	48,5
[20 - 25 [	29	14,2
[25 - 30 [	9	4,4
[30 - 35 [	3	1,5
**Total**	**204**	**100**

### Âge au moment de la pratique de la mutilation génitale et nombre de mutilations

L’âge des patientes au moment de la pratique de la mutilation a été précisé chez 156 patientes, soit 86,3 %. Cet âge variait entre 1 an et 15 ans. Le Tableau II représente la répartition selon la tranche d’âge des patientes au moment de la mutilation.

**Tableau II:** Répartition selon la tranche d’âge au moment de la mutilation, des patientes prises en charge pour une complication de mutilation génitale féminine au CHU régional de Ouahigouya de 2009 à 2022 Distribution by age group at the time of mutilation, of patients treated for a complication of female genital mutilation at the Ouahigouya regional teaching hospital from 2009 to 2022

Âge (années)	Effectif	Pourcentage
[00 - 05[	28	18
[05 - 10 [	98	62,8
[10 - 15 [	25	16
[15 - 20 [	5	3,2
**Total**	**204**	**100**

Après la cicatrisation d'une première mutilation, 11 patientes ont été mutilées une 2^e^ fois. Parmi ces dernières, 3 patientes ont été à nouveau mutilées une troisième fois.

### Caractéristiques socio-professionnelles des patientes

Le Tableau III présente les caractéristiques socio-professionnelles des patientes.

**Tableau III:** Répartition selon les caractéristiques socio-professionnelles des patientes prises en charge pour une complication de mutilation génitale féminine au CHU régional de Ouahigouya de 2009 à 2022 Distribution according to socio-professional characteristics of patients treated for a complication of female genital mutilation at the Ouahigouya regional teaching hospital from 2009 to 2022

Caractéristiques socio-professionnelles	Fréquence	Pourcentage
Statut matrimonial		
mariée	73	35,8
célibataire	52	25,5
non concernée*	79	38,7
**Total**	**204**	**100**
Résidence		
zone urbaine	108	52,9
zone rurale	96	47,1
**Total**	**204**	**100**
Profession		
élève/écolière	66	32,4
ménagère	104	51
salariée	7	3,4
sans emploi	12	5,9
non concernée*	15	7,4
**Total**	**204**	**100**

* Pour la variable statut matrimonial, la modalité « non concernée » désigne les patientes âgées de moins de 18 ans non mariées. Pour la variable profession, elle désigne les patientes qui nont pas encore 6 ans, l’âge de scolarisation obligatoire au Burkina Faso.

Motifs de consultation et itinéraire des patientes

Les motifs de consultation des patientes sont représentés dans le Tableau IV.

**Tableau IV:** Répartition selon le motif de consultation des patientes prises en charge pour une complication de mutilation génitale féminine au CHU régional de Ouahigouya de 2009 à 2022 (n = 204) Distribution by reason for consultation of patients treated for a complication of female genital mutilation at the Ouahigouya regional teaching hospital from 2009 to 2022 (n=204)

Motif de consultation	Effectif	Pourcentage
Rapports sexuels impossibles	90	44,1
Rétrécissement vulvaire*	83	40,7
Dysurie**	64	31,4
Dyspareunie	17	8,3
Plaie génitale	14	6,9
Douleurs pelviennes intermittentes	11	5,4
Tuméfaction génitale	8	3,9
Dysménorrhée	6	2,9

* Parmi les patientes ayant consulté pour un rétrécissement vulvaire, il y avait 13 femmes enceintes, dont 6 étaient en travail d'accouchement.

** Ce motif a été observé seulement chez les patientes de moins de 15 ans.

Les patientes étaient référées d'une formation sanitaire périphérique dans 33,3 % des cas (68 patientes). Elles venaient d'elles-mêmes dans 30,9 % des cas (63 patientes), étaient recommandées par une association de lutte contre les mutilations génitales féminines dans 28,9 % (59 patientes), ou adressées par les services de l'action sociale dans 6,9 % des cas (14 patientes).

Le délai entre la mutilation et la consultation s’étendait de 10 jours à 21 ans pour les patientes qui se souvenaient du moment de la mutilation.

### Diagnostics retenus

Après l'examen clinique, la mutilation génitale était de type III chez 187 patientes soit 91,8 %, et de type II chez 18,2 %. Les complications diagnostiquées sont présentées dans le Tableau V. Plusieurs diagnostics étaient souvent associés chez une même patiente.

**Tableau V:** Répartition selon le diagnostic, des patientes prises en charge pour une complication de mutilation génitale au CHU régional de Ouahigouya de 2009 à 2022 Distribution according to diagnosis, of patients treated for a complication of genital mutilation at the Ouahigouya regional teaching hospital from 2009 to 2022

Diagnostics retenus	Fréquence	Pourcentage
**Mutilations génitales de type III (n = 187)**		
complications immédiates	0	0
complications à moyen et long termes	187	100
apareunie et dyspareunie	123	65,8
dysurie	64	34,2
dystocie d'expulsion	13	7
chéloïde	8	4,3
dysménorrhée	6	3,2
urocolpos et reflux d'urine dans la cavité utérine	4	2,1
infection urinaire	4	2,1
synéchie vaginale	1	0,5
**Mutilation génitale de type II (n = 17)**		
complications immédiates	0	0
complications à moyen et long terme	17	100
infection du site de la mutilation	14	82,4
chéloïde	3	17,7
dyspareunie	3	17,7

### Prise en charge et évolution

Les complications diagnostiquées ont motivé un traitement chirurgical chez 189 patientes (92,7 % des patientes), soit toutes les patientes ayant eu une infibulation et 2 patientes ayant eu une mutilation de type II. Un traitement médicamenteux seul a été administré à 15 autres patientes (7,4 % des patientes) ayant eu une mutilation de type II.

Pour les 189 patientes opérées, 116 soit 61,4 % ont bénéficié d'une anesthésie générale ou d'une rachianesthésie, et 73 soit 38,6 % d'une anesthésie locale à la xylocaïne à 1 %. Parmi les patientes ayant bénéficié d'une anesthésie générale ou rachianesthésie, on dénombrait 30 patientes chez qui une anesthésie locale avait été initialement prévue.

Les gestes chirurgicaux réalisés sont représentés dans le Tableau VI.

**Tableau VI:** Répartition selon le geste chirurgical réalisé, des patientes opérées pour une complication de mutilation génitale féminine, au CHU de Ouahigouya du 2009 au 2022 (n = 189) Distribution according to the surgical procedure performed, of patients operated for a complication of female genital mutilation, at the Ouahigouya regional teaching hospital of from 2009 to 2022 (n = 189)

Geste chirurgical	Fréquence	Pourcentage
Ablation de chéloïde	10	5,3
Désinfibulation*	187	98,9
Cure de synéchie vaginale	1	0,5
Méatoplastie	1	0,5

* La désinfibulation a été réalisée chez 7 patientes au cours d'une grossesse, chez 6 patientes au cours de l'accouchement.

L’évolution sous traitement a été favorable dans tous les cas. Les 13 femmes enceintes désinfibulées ont accouché par voie basse, avec cependant une épisiotomie chez 10 d'entre elles.

## Discussion

### Épidémiologie

Dans notre étude, nous avons trouvé une fréquence annuelle de 15,7 complications de mutilations génitales féminines qui semble relativement faible par rapport à d'autres études. En effet, Akotionga [1] avait colligé 49 cas en 1998 à Ouagadougou au Burkina Faso, au cours d'une période où peu de centres hospitaliers prenaient en charge les complications des mutilations génitales dans ce pays. Dembélé [4] en 2018 à Kayes au Mali a enregistré 77 complications en 2 ans, soit une fréquence annuelle de 38,5.

Notre fréquence est en deçà de la réalité car nous n'avons pas pu inclure tous les types de complications. Ainsi les états de stress post-traumatiques n'ont pas été systématiquement recherchés parce que les équipes de prise en charge comprenaient rarement un professionnel de la santé mentale, et n’étaient pas formées à reconnaître ces états. Chez les parturientes ayant une mutilation des types I et II, l'absence d'informations permettant d'incriminer la mutilation dans la survenue d'une épisiotomie ou d'une déchirure ne nous ont pas permis d'inclure ces complications. Enfin les absences ou diminutions du désir et du plaisir sexuels chez la femme mutilée n'ont pas été recensées parce que non abordées par les soignants, ni énoncées par les patientes du fait d'une certaine pudeur dans notre contexte burkinabè, qui semble interdire d’évoquer le plaisir et le désir sexuels.

On s'attendait donc à dénombrer plus de complications dans notre étude, car les mutilations génitales féminines sont encore fréquentes dans la région du Nord du Burkina Faso, avec 76 % de femmes mutilées [10]. Ces pratiques sont si ancrées dans les coutumes que les jeunes filles, comme certaines de notre étude, peuvent être soumises deux, voire trois fois au rituel douloureux, jusqu’à l'excision intégrale du clitoris et souvent des petites et grandes lèvres. De plus, comme le montrent nos résultats et ceux d'autres études [1, 4], les victimes porteuses de complications peuvent attendre plusieurs années avant de consulter. Dans notre contexte, avec la loi interdisant la pratique de la mutilation génitale féminine au Burkina Faso, la crainte de devoir faire face à la justice amène à ne pas consulter, surtout en cas de complications survenant dans les suites plus ou moins immédiates de la pratique. C'est ainsi que dans notre étude, aucune complication immédiate n'a fait l'objet de consultation. Les cas d'infection du site mutilé que nous avons recensés et qui relèvent de complications à moyen terme ont fait l'objet de consultations parce que ces cas ont été dénoncés auprès des services de l'action sociale qui ont conduit les victimes à l'hôpital pour une prise en charge.

Les associations de lutte contre la pratique des mutilations génitales ont été déterminantes dans la prise en charge de certaines patientes. En effet, en plus de sensibiliser pour l'abandon de la pratique des mutilations génitales, elles informent également les populations des possibilités de prise en charge médicale des complications, et facilitent l'accès des victimes aux soins.

La tranche d’âge de 15 à 24 ans a été la plus représentée dans notre population d’étude, de même que dans celles d'Akotionga [1] et Dembélé [4]. C'est dans cette tranche que l'on retrouve l’âge de 17 ans, la plus représentée. Cet âge est proche de l’âge médian aux premiers rapports sexuels qui est de 17,4 ans et de l’âge médian à la première union qui est de 17,7 ans dans la région du Nord du Burkina Faso [6]. D'ailleurs, parmi les patientes de notre étude, il y avait une grande proportion de femmes mariées. Ainsi dans cette tranche d’âge, les jeunes filles qui ont pour la plupart été mutilées à l'enfance et qui ont présenté un rétrécissement vulvaire sans autres symptômes gênants, se trouvent dans l'impossibilité de pratiquer des rapports sexuels ou se trouvent confrontées aux dyspareunies ou aux dystocies [1], d'où la recherche de soins. En plus des troubles sexuels, la dysurie a été un motif de consultation fréquent surtout chez les enfants, dans notre étude. C'est le constat fait également dans certaines études [2, 4, 15].

### Aspects cliniques

Les complications infectieuses dans notre étude sont le reflet des conditions dans lesquelles les mutilations génitales féminines sont pratiquées. En effet la pratique est clandestine, sans respect des principes de prévention et de contrôle de l'infection, avec des instruments non adaptés et souvent utilisés pour plusieurs personnes à la fois. Tous les germes peuvent être retrouvés dans ces conditions. Cependant cela n'est pas une raison pour promouvoir la médicalisation de la pratique dans le but d'améliorer les conditions d'hygiène [3, 12]. En effet, le risque de survenue des autres complications ne peut être prévenu avec la médicalisation, et les mutilations demeurent une atteinte aux droits de la femme à l'intégrité physique et à la santé. Fort heureusement cette médicalisation qui est peu pratiquée dans notre contexte burkinabè [10], est punie par le code pénal du Burkina Faso de 1997 révisé en 2018 pour rendre les sanctions plus pénibles. Nous n'avons pas eu de cas de complications immédiates telles que l'hémorragie et la douleur, contrairement à Dembélé à Kayes au Mali où les mutilations génitales féminines ne sont pas punies par la loi [5]. Dans notre contexte, la pression de la loi punissant les mutilations génitales de peines de prison et d'amendes pourrait décourager le recours aux structures de soins en cas d'hémorragies importantes ou d'autres complications immédiates. Il faut alors craindre que certaines filles décèdent des suites de certaines complications immédiates de ces pratiques clandestines.

La plupart de ces complications étaient survenues (81,8 % des cas) avec les mutilations génitales de type infibulation, bien que ce type de mutilation ne soit pas le type le plus pratiqué dans notre pays [7]. C'est bien parce que l'infibulation est la plus grande pourvoyeuse de complications [11] que sa fréquence dans notre étude est importante.

Parmi les complications observées, l'urocolpos et le reflux d'urine dans l'utérus dans le contexte de mutilations génitales féminines ont été rarement rapportés dans la littérature [13]. Dans notre cas, ils ont été des découvertes échographiques fortuites. Cependant, le contexte clinique dans lequel ils ont été découverts était évocateur, marqué par des douleurs pelviennes intermittentes, une dysurie de poussée, des épisodes de rétention d'urine, une infibulation avec un orifice vulvaire punctiforme identifiable seulement lors des mictions qui se font goutte à goutte et une vulve rénitente en regard de l'orifice vaginal. L'urocolpos et le reflux d'urine dans l'utérus peuvent se compliquer d'infection urinaire, de septicémie et d'uropéritoine.

### Prise en charge et évolution

La prise en charge des patientes a nécessité un traitement chirurgical dans la majorité des cas. La désinfibulation a été le geste chirurgical le plus réalisé. Elle a suffi à corriger la plupart des complications. Aucune reconstruction du clitoris n'a été réalisée au cours de la prise en charge chirurgicale. Cela s'explique d'une part par le fait que les chirurgiens n’étaient pas formés à la technique de reconstruction clitoridienne jusqu'en 2015. Après cette date, les patientes adultes qui ont reçu l'information de la possibilité d'une reconstruction clitoridienne au cours de leur préparation, n'en ont pas fait la demande, probablement à cause des représentations sociales autour des parties mutilées.

La chirurgie a été réalisée sous anesthésie locale dans seulement 38,6 % alors que l'OMS [11] recommande l'anesthésie locale lors de la désinfibulation qui a été le geste chirurgical le plus réalisé chez nos patientes. Dans notre cas, tous les enfants ont bénéficié d'emblée d'une anesthésie générale, car difficiles à maîtriser pour une procédure chirurgicale avec anesthésie locale. Parmi les adolescentes et les adultes, une fois informées sur la procédure chirurgicale et l'anesthésie au cours de leurs préparations, un certain nombre a opté d'emblée pour une anesthésie générale ou une rachianesthésie. D'autres qui avaient choisi l'anesthésie locale ont présenté des réflexes de défense et une certaine agitation lors de l'infiltration de la xylocaïne, amenant à leur administrer plutôt une anesthésie générale ou une rachianesthésie. Comme certains auteurs [1, 14], nous avons pensé que ces patientes pourraient avoir eu tendance à revivre la scène de leur mutilation, et à développer de ce fait un réflexe de défense et une certaine agitation malgré toute la préparation préopératoire.

Les traitements des complications des mutilations génitales sont en général efficaces pour soulager les victimes des signes physiques génito-urinaires [9]. Pour les femmes enceintes, la désinfibulation en cours de grossesse ou pendant l'accouchement rend possible l'accouchement par voie basse au prix, souvent, d'une épisiotomie pour réduire le risque de déchirures graves et compliquées du périnée [8].

## Conclusion

Les mutilations génitales féminines sont une source de complications à l'origine de handicaps pour la vie sexuelle et reproductive des victimes. De nombreuses complications ont certes été prises en charge au Centre hospitalier universitaire régional de Ouahigouya de 2009 à 2022, mais la fréquence de ces complications pourrait être encore plus importante, car la pratique des mutilations génitales est répandue dans la région du Nord du Burkina, et le recours aux soins appropriés tardif. Heureusement, la prise en charge de ces complications est efficace et permet de soulager les victimes de leurs lésions physiques. En revanche leurs souffrances psychologiques restent à être évaluées et prises en charge suite à ces mutilations qui constituent une violation des droits humains. Il reste aussi à espérer que les victimes prises en charge ne reproduisent pas sur leurs fillettes ces mutilations génitales et adoptent une attitude favorable à l'abandon de cette pratique dans leurs entourages. La cure chirurgicale devrait être l'occasion de campagnes d’éducation sanitaire personnalisées visant leur entourage familial féminin et masculin, ascendants et collatéraux en arguant à la fois des souffrances physiques et psychologiques infligées sans consentement des victimes, des conséquences sur la santé et aussi des risques pénaux.

## Principes éthiques

La direction des services médicaux et techniques du Centre hospitalier universitaire régional de Ouahigouya qui a en charge les questions éthiques, a donné son accord pour notre étude.

## Rôles et accords des auteurs

Sansan Rodrigue SIB, Évelyne KOMBOÏGO et Moussa SANOGO ont conçu le protocole, recueilli les données et rédigé la première version du manuscrit.

Issa OUÉDRAOGO, Alexandre TARNAGADA, Souleymane TRAORÉ et Charlemagne Marie Ragnag-Néwendé OUÉDRAOGO ont apporté des contributions essentielles au protocole et au manuscrit. Tous les auteurs ont lu et approuvé le document final.

## Conflits d'intérêts

Les auteurs déclarent ne pas avoir de lien d'intérêts en rapport avec cet article. Cette étude n'a pas bénéficié de financement.
